# Urologic practice patterns of pediatricians: a survey from a large multisite pediatric care center

**DOI:** 10.3389/fped.2023.1278782

**Published:** 2023-12-06

**Authors:** Courtney A. Stewart, Soo Jeong Kim, Daniel Phillips, Vinaya Bhatia, Nicolette Janzen, Jonathan A. Gerber

**Affiliations:** ^1^ Division of Urology, Department of Surgery, University of Texas Medical Branch, Galveston, TX, United States; ^2^ Division of Urology, Columbia University School of Medicine, New York, NY, United States; ^3^ Division of Urology, UW School of Medicine and Public Health, Madison, WI, United States; ^4^ Division of Pediatric Urology, Department of Surgery, Texas Children's Hospital, Houston, TX, United States

**Keywords:** pediatrician, vesicoureteral reflux, undescended testis, urinary tract infection, referral, practice pattern

## Abstract

**Objective:**

To evaluate the practice patterns of pediatricians as they relate to common urologic concerns.

**Materials and methods:**

An anonymous 15-question survey was created and distributed to all pediatricians at our institution, a large multisite care center. This study was deemed exempt by the institutional review board.

**Results:**

55 of the 122 (45%) providers queried responded. 93% of the participants were female, and 7.3% were male. 55% recommended testicular self-examination at adolescence, while 39% did not recommend at any age. 78% stated that they were “Fairly confident” in the exam for undescended testicle (UTD). One-third referred patients with UDT to a subspecialist upon recognition at birth, 13% at 3 months of age, and 28% at 6 months of age. 10% reported obtaining a VCUG after the first febrile urinary tract infection (UTI), 26% after the second, and 36% only if there were abnormal findings on renal ultrasound. 28% of providers reported that they refer to pediatric urology after the initial febrile UTI. 19% provided antibiotics for UTI symptoms alone with negative urinalysis and urine culture.

**Conclusions:**

Despite established guidelines, practice patterns varied among pediatricians. Pediatricians typically followed the AAP's guidelines regarding VCUGs (62%), with only a few adhering to urologic recommendations (9%). Despite the consistency between AAP and AUA guidelines regarding the age at which to refer a patient for cryptorchidism, about 70% of practitioners referred patients too early or too late. Harmonized, consolidated guidelines between pediatricians and pediatric urologists would improve patient care and efficiency of the healthcare system.

## Introduction

Management of surgical conditions in children by board certified pediatric surgical specialists have been associated with improved outcomes (1–3). As such, the American Academy of Pediatrics (AAP) has created referral recommendations to assist general pediatricians in determining when, and to whom, to refer their patients for pediatric surgical specialty care (4). The AAP recommends referral to a pediatric urologist for a variety of urologic conditions including undescended testes, congenital hydroceles and hernias, hypospadias, solid malignancy of a genitourinary organ, and moderate or severe vesicoureteral reflux.

Despite these guidelines, the decision to refer a child to a pediatric urologist can be complex. From the pediatrician's perspective, potential barriers can include long wait times for appointments with pediatric surgical specialties and surgical subspecialists' nonparticipation in health insurance plans (5). To further complicate matters, the AAP guidelines can differ from specialty-specific guidelines regarding the same pathology. For instance, while the most recent AAP guidelines recommends against a routine voiding cystourethrogram (VCUG) after the first febrile urinary tract infection (UTI) unless other risk factors are present (6), the Section on Urology of the AAP expressed significant concern regarding this recommendation as it could delay the diagnosis of harmful urinary tract conditions (7).

In addition, inappropriate referrals can consume a significant amount of health-system resources. A recent retrospective review showed that 19% of pediatric surgery referrals are inappropriate, and that children traveled an average of 57.4 miles for an inappropriate referral with a pediatric surgeon, with the travel burden being even more pronounced in children who lived in rural communities (8).

In this study, we sought to better understand the practice patterns for common urologic conditions of general pediatricians in our large network of community based pediatricians, including referral to pediatric urologists, in order to identify potential areas for improvement.

## Methods

An online survey was developed and distributed to all pediatricians at our institution. The questions targeted pediatrician practice patterns for common urologic conditions. Topics included the frequency of performance of genitourinary (GU) exams, referral patterns for undescended testicles (UDT), recommendation for testicular self-examination, work up for urinary tract infections (UTI) including additional imaging (i.e., VCUG), and antibiotic stewardship in common UTI scenarios (Table 1). There were 15 questions in the survey; each question was multiple choice and was independent of all other questions in the survey. All responses were anonymous. The survey took respondents approximately 2 min to complete.

**Table 1:** Survey questions and answers.

Q1. How many years have you been practicing in pediatrics?		
ANSWER CHOICES	RESPONSES	
0–5 years	12	21.8%
6–10 years	12	21.8%
11–15 years	8	14.5%
16–20 years	3	5.5%
20 + years	20	36.4%
Total	55	
Q2. What is your educational background?		
ANSWER CHOICES	RESPONSES	
MD/DO	45	81.8%
PA	3	5.5%
NP	7	12.7%
Total	55	
Q3. What is your gender?		
ANSWER CHOICES	RESPONSES	
Male	4	7.3%
Female	51	92.7%
Nonbinary	0	0%
Other	0	0%
Total	55	
Q4. General: How often do you perform a comprehensive genitourinary (GU) examination for female patients (well-child)?		
ANSWER CHOICES	RESPONSES	
GU examination is never performed at a well-child visit unless the patient or guardian(s) report a concern	2	3.8%
GU examination is performed at every well child visit, but stopped at adolescence	21	40.4%
GU exam is always performed at all ages and every well-child visit	21	51.9%
GU examination is only performed at initial visit and if normal, forego future examinations	2	3.8%
If patient sees urology, I do not continue to perform GU examinations	0	0%
Total	52	
Q5. General: How often do you perform a comprehensive genitourinary (GU) examination for male patients (well-child)?		
ANSWER CHOICES	RESPONSES	
GU examination is never performed at a well-child visit unless the patient or guardian(s) report a concern	1	1.9%
GU examination is performed at every well child visit, but stopped at adolescence	9	17.0%
GU exam is always performed at all ages and every well-child visit	40	75.5%
GU examination is only performed at initial visit and if normal, forego future examinations	3	5.7%
If patient sees urology, I do not continue to perform GU examinations	0	0%
Total	53	
Q6. General: Do you recommend testicular self-examination?		
ANSWER CHOICES	RESPONSES	
Yes	33	61.1%
No	21	38.9%
Total	54	
Q7. General: If you recommend testicular self-examination, at what age do you recommend it to patients?		
ANSWER CHOICES	RESPONSES	
Testicular self-examination is recommended at any age	1	2.0%
Parental testicular exam is recommended starting after birth	0	0.0%
Testicular self-exam is recommended once the patient demonstrates understanding	5	9.8%
Testicular self-exam is recommended beginning at adolescence	28	54.9%
Testicular self-exam is not recommended	17	33.3%
Total	51	
Q8. General: If you recommend testicular self-examination, how do you teach your patients to properly perform exam?		
ANSWER CHOICES	RESPONSES	
Verbally tell patients how to perform exam	14	26.9%
Demonstrate to patients how to perform exam	11	21.2%
Give patients literature/pamphlet to review	3	5.8%
No particular teaching is given regarding testicular self exam	7	13.5%
Testicular self-exam is not recommended	17	32.7%
Total	52	
Q9. UTI: At what time point do you order a VCUG following a febrile UTI?		
ANSWER CHOICES	RESPONSES	
After the first febrile UTI	5	9.4%
After the second febrile UTI	14	26.4%
Only if there are abnormal findings on renal ultrasound	19	35.8%
After the first febrile UTI, referral to pediatric urology for evaluation and initiation of workup	15	28.3%
Total	53	
Q10. UTI: Which of the following would you define as a UTI? (Select all that apply)		
ANSWER CHOICES	RESPONSES	
Positive urine dipstick alone	2	3.7%
Positive culture alone	19	35.2%
Smelly urine alone	2	3.7%
Positive microscopic urinalysis, positive culture, and symptoms (frequency, urgency, dysuria, SP pain, etc)	44	81.5%
Total	54	
Q11. UTI: If a patient is having symptoms suggestive of a UTI (e.g. dysuria, frequency, urgency, suprapubic discomfort) but the urinalysis and urine culture are negative, will you prescribe antibiotics to treat a presumed UTI?		
ANSWER CHOICES	RESPONSES	
Yes	10	18.9%
No	43	81.1%
Total	53	
Q12. UTI: In patients with new onset UTI, do you assess patients for concurrent constipation?		
ANSWER CHOICES	RESPONSES	
Yes	45	83.3%
No	9	16.7%
Total	54	
Q13. Undescended Testicle (UDT): On male GU examination, how confident are you in the exam for undescended testicle?		
ANSWER CHOICES	RESPONSES	
Completely confident	6	11.1%
Fairly confident	42	77.8%
Somewhat confident	4	7.4%
Slightly confident	2	3.7%
Not confident at all	0	0.0%
GU exam is not routinely performed	0	0.0%
Total	54	
Q14. UDT: Upon clinical suspicion for undescended testicle, what is your next step?		
ANSWER CHOICES	RESPONSES	
Referral	25	45.5%
Repeat exam in 6 months	7	12.7%
Obtain ultrasound	23	41.8%
Total	55	
Q15. UDT: For a child with undescended testicle, at what age do you provide referral to pediatric urology/surgery?		
ANSWER CHOICES	RESPONSES	
At birth/Immediately upon recognition	16	29.7%
3 months of age	4	7.4%
6 months of age	15	27.8%
9 months of age	6	11.1%
1 year	13	24.1%
Puberty	0	0.0%
No referral to pediatric urology/surgery	0	0.0%
Total	54	

Responses were analyzed quantitatively and evaluated for adherence to various existing guidelines (American Urological Association [AUA], American Academy of Pediatricians [AAP]).

As the survey was anonymous and confidential (no identifying information was collected with the survey), this study posed minimal risk to the participants and was exempt from a full Institutional Review Board committee review.

## Results

55 of the 122 providers queried responded for a response rate of 45.0%. 92.7% of the respondents completed the survey in its entirety. The largest cohort of those surveyed, 36.4%, had been practicing in pediatrics for over 20 years. 21.8% have practiced less than 5 years, 21.8% have been practicing 6–10 years, 14.5% have been practicing 11–15 years, and 4.5% have been in practice for 16–20 years. 81.8% of the participants were MD/DO trained, 12.7% were nurse practitioners (NPs), and 5.5% were physician assistants (PAs). 92.7% of the participants were female, and 7.3% were male. All survey questions and answers are outlined in Table 1.

52% of participating providers reported that they perform comprehensive GU exams at each female well-child visit. 40% percent reported stopping routine GU exams once the female reached adolescence. Two providers did not perform a GU exam unless the parents reported a concern, and two providers performed GU exam and if normal, would forego future exams. 75.5% of providers performed a comprehensive GU exam for male patients at all ages and every well child visit. 17% reported that they stopped performing GU exams for males at adolescence. Three providers would perform GU exam at the initial visit and forego future exams if normal for male patients. One provider only performed GU exam for male patients if the parents mentioned a problem.

Testicular self-examination was recommended by the majority of responding providers (61.1%), but the timing of the counseling varied: 55% of providers recommended commencing testicular self-examination at adolescence, ten percent once the patients demonstrated understanding, and one percent recommended testicular self exam at any age. 39% did not recommend testicular self-examination. In addition, providers varied in their teaching method for proper testicular exam. 27% verbally told patients how to perform exam, and 21% demonstrated to patients how to perform the exam. 5.8% gave patients literature to review, and 13.5% did not provide any specific training.

The majority of respondents, 78%, stated that they were “Fairly confident” in the exam for undescended testicles. 11% stated they were “completely confident”, while seven percent said they were “somewhat confident” and four percent said they were “slightly confident”. At the time of clinical suspicion for an undescended testicle, 45.5% of providers said they would refer and 42% said they would obtain an ultrasound. 13% of providers said they would repeat the exam in six months.

Roughly one-third of respondents referred a patient to pediatric urology or pediatric surgery for undescended testis at the time of recognition at birth. Additionally, 7.4% preferred to refer at 3 months of age and 28% at 6 months of age. 11% referred the patient for surgical consultation at 9 months and 24.1% referred at one year of age. No providers reported delaying referral until puberty.

After initial febrile UTI, 10% of respondents reported ordering a VCUG while 28% report that they would directly refer to pediatric urology for all further work up. The largest group of providers (36%) ordered a VCUG only if there were abnormal findings on renal ultrasound. 26.4% preferred to order a VCUG after a second febrile UTI.

In the setting of a negative urinalysis and urine culture, 19% of respondents stated they would provide antibiotics for UTI symptoms (e.g., dysuria, frequency, urgency, suprapubic discomfort) alone. The majority of providers (81.5%) stated that they would define positive microscopic urinalysis, positive culture, and symptoms (frequency, urgency, dysuria, SP pain, etc) as a UTI. 35% of providers stated that positive urine culture alone would be a UTI. 3.7% stated they would define smelly urine as a UTI and 3.7% stated they would define positive urine dipstick alone as a UTI. In patients with new onset UTI, 83% did assess patients for concurrent constipation.

## Discussion

**Figure 1:**
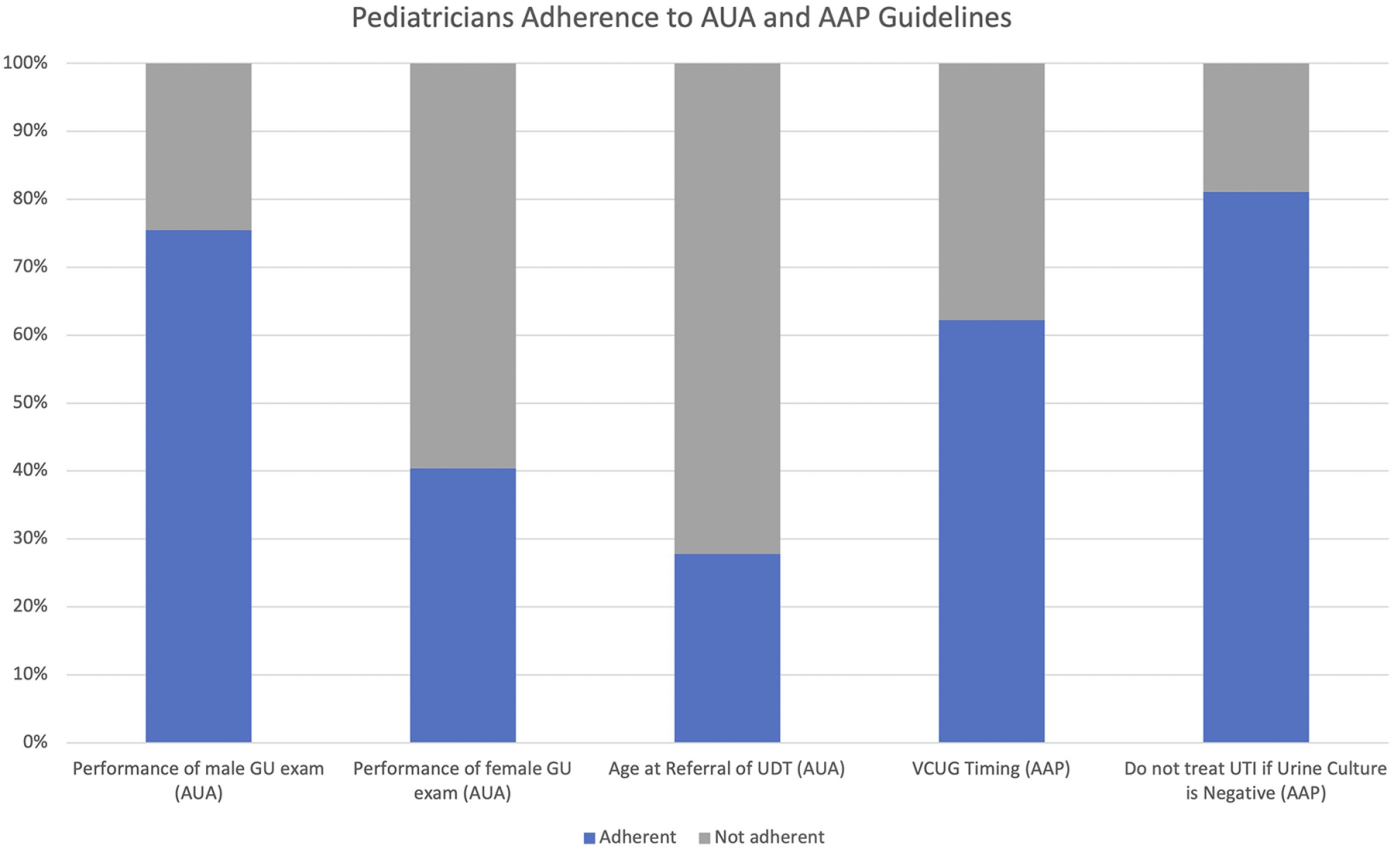
Pediatricians’ adherence to AUA and AAP guidelines. This figure represents the percentage of pediatricians who adhere to AUA and AAP guidelines. 75% of pediatricians adhered to AUA standards regarding the performance of GU exam. 40% of pediatricians adhered to AUA standards regarding performance of female GU exam. 28% of pediatricians adhered to AUA guidelines regarding the timing of referral for UDT. 62% of pediatricians adhered to the AAP guidelines regarding VCUG timing. 81% of pediatricians adhered to AAP guidelines regarding treating UTls with a negative urine culture.

This study highlighted notable discrepancies in practice patterns by pediatricians when managing urologic conditions. Starting with the GU physical exam, the majority (75.5%) of survey participants indicated that they perform one at every visit for male children regardless of age, which is less than a previous study that reviewed GU examination documentation in well-child visits where over 95% had a documented GU exam (Figure 1) (9). Interestingly, 17% indicated they stopped GU examinations for males at adolescence, which is consistent with the United States Preventive Services Task Force (USPSTF)'s recommendation against routine testicular examinations in asymptomatic adolescents and adults for the purposes of testicular cancer screening (10). A comprehensive GU exam, however, can also identify other pathologies that present later in childhood and require surgical intervention, such as testicular ascent. A retrospective review of patients who underwent late orchiopexies, defined as at 4 years of age or later, showed that the most common reasons for late intervention were ascending testis (45%), parental delay (22%), and late referral (20%) (11). As such, an annual GU exam is recommended by the AUA (12). In addition, although the survey questions were designed to capture broad practice patterns and did not address such subtleties, a pediatric patient with a history of cryptorchidism should be monitored closely for testicular cancer given their higher risk, regardless of age (12). Also of note, providers indicated different practice patterns for females, with 52% of providers performing GU exams for female patients at all ages and 40% of providers stopping at adolescence. This discrepancy may be due to need to monitor for complications such as ascending testes or testicular masses. It could also be due to the expectation that some young women may begin visits with gynecologists at puberty; the American College of Obstetricians and Gynecologists recommends an initial reproductive health visit with an OBGYN between the ages of 13 and 15 (13).

Relatedly, approximately 60% of surveyed providers recommended testicular self-examination, with the majority (55%) having their patients initiate self-examinations beginning at adolescence. Additionally, there was great discrepancy regarding how to teach patients to perform testicular self-examination, with 27% verbally teaching and 22% demonstrating how to perform the exam. It is possible that the providers who ceased performing in-office GU examinations once patients reached adolescence were more reliant on the patients performing testicular self-examinations. This approach should be undertaken with caution; in one study from the Netherlands, it was shown that a mere 2% of teenagers routinely perform testicular self-examinations (14).

For pediatric patients with clinical suspicion for undescended testicle, 45.5% of providers stated they referred the patient to urology and 42% of providers obtained an ultrasound even though routine use of ultrasound has been specifically advised against by AUA guidance due to an estimated sensitivity and specificity of 45% and 78%, respectively (15). Furthermore, only 11% of pediatricians indicated that they were “completely confident” in their exam for undescended testicle and 78% indicated that they were “fairly confident.” Slightly lower confidence levels may lead to unnecessary referrals for UDT or in contrast, it may mean pediatricians miss an UDT on exam and fail to make the appropriate referral at six months. AUA guidelines recommend pediatric exams for undescended testicles until 6 months of age, at which time referral is required to evaluate if descent fails to occur (12).

For a pediatric patient with identified cryptorchidism, only 28% of providers referred patients to a specialist at 6 months of age, despite the similar AAP and AUA guidelines regarding timing of referral and surgical correction (Figure 1) (12, 16). Nearly a third of providers (30%) referred their patients to a surgical specialist for cryptorchidism immediately upon recognition at birth and 7.4% at 3 months of age despite previous studies showing that 35%–80% of newborn boys followed longitudinally have spontaneous descent of congenitally cryptorchid testes, usually prior to 3 months of age (15, 17, 18). In other words, almost half of the patients referred for UDT at birth could have received an unnecessary surgical subspecialist consult. On the other end of the spectrum, 11% of providers delayed referral until 9 months of age and 24% of providers delayed referral until 1 year of age which is inconsistent with known low rates of spontaneous testicular descent after 6 months of age. The AUA guidelines recommend surgical correction between 6 and 18 months of age; a delayed referral after 1 year of age, when accounting for time to schedule an evaluation with a pediatric urologist and then a surgery if necessary, could push the timing of surgical correction past this critical time frame.

The timing of obtaining a VCUG has historically been controversial and our study suggests that this continues to be an area of ongoing debate. 62% of pediatricians (36% obtained if abnormality seen in ultrasound and 26% obtained after second febrile UTI) followed the most recent guidelines put forth by the AAP, which recommends against a routine VCUG after the first febrile UTI unless abnormalities are present on the renal bladder ultrasound or there are other clinical reasons to suspect high-grade vesicoureteral reflux or obstructive uropathy (Figure 1) (6). Although the majority of pediatricians are following AAP guidelines in terms of timing of obtaining a VCUG, there is growing evidence that renal scarring may be missed by not obtaining VCUGs after the first UTI: Narchi et al. showed that following AAP guidelines would have missed 56% of children with VUR ≥ grade II, and all children with renal scarring would not have been imaged (19). In addition, when cross-sectional cohorts of patients in 2005 and 2015 were compared, patients presenting in 2015 for post-UTI VCUG had an increased likelihood of recurrent UTI and renal scarring (17). The AAP and EAU/ESPU guidelines recommend referral after first febrile UTI in part due to a study which evaluated severity of renal scarring after febrile UTIs (6, 20). The study illustrated 3% after the first UTI which then increased to 26% after the second, indicating a critical period between the first and second UTI (21). While VCUGs are an invasive imaging modality and can cause morbidity such as patient and parent distress, although perhaps less with pretest preparation and child life specialist involvement (22), future studies will be necessary to delineate risk factors for developing renal scarring in order to not improperly delay the diagnosis of symptomatic vesicoureteral reflux.

A small number (9%) of pediatricians did prefer to order a VCUG after the first febrile UTI, which is supported by the Section of Urology (7). Another interesting finding was approximately 28% of pediatricians referred patients to pediatric urology for evaluation following the first febrile UTI without further investigation, which could be an area for collaboration in order to reduce unnecessary referrals.

In regards to antibiotic use in a patient with symptoms suggestive of UTI/cystitis, the majority of providers (81%) stated that they would not prescribe antibiotics in the setting of a negative urinalysis and urine culture, which is in line with the AAP guidelines recommending both urinalysis results that suggest infection and the presence of at least 50,000 colony-forming units (CFU) per mL on a urine culture obtained in order to establish the diagnosis of UTI (Figure 1) (6). Nearly 19% of respondents stated that they would prescribe antibiotics in this clinical setting, however, which is concerning as antibiotic usage is not benign, particularly if not indicated: early exposure to antibiotics in childhood could have a negative effect on neurocognitive function, body metabolism, and increased resistance to common antimicrobial agents (23).

Although almost 82% of providers defined positive microscopic urinalysis, positive culture, and symptoms as a UTI, 35% also defined positive urine culture alone as a UTI. It is important to note that the goal of UTI therapy is renal preservation, and isolated bacteriuria without symptoms does not warrant treatment as asymptomatic bacteriuria is often self-resolving (24). Only 3% of providers defined smelly urine alone as a UTI, consistent with the current understanding that malodorous or cloudy urine has been shown to be an unreliable marker for UTI (25). In the absence of clear evidence-based protocols, it is likely that many patients are being overtreated with antibiotics.

The goal of this study was to establish broad practice and referral patterns of the general pediatricians in regards to management of urologic conditions. In order to maximize participation, the survey questions were not designed to portray the various nuances that could be present in a real-life clinical encounter, which is a limitation of this study. The providers were also asked to only select one answer choice that best represented their predominant practice preference, but they might have chosen other answers in different clinical scenarios. In addition, perhaps as a reflection of the online format (26), the overall participation rate by the pediatricians was low although in line with reported response rates for many surveys.

## Conclusion

Pediatricians typically followed the AAP's guidelines regarding VCUGs (62%), with only a few adhering to urologic recommendations (9%). Despite the consistency between with AAP and AUA guidelines regarding the age at which to refer a patient for cryptorchidism, about 70% of practitioners referred patients too early or too late. Despite a lack of supportive guidelines, most providers did recommend testicular self-examination at some point. Most providers (81%) stated that they would not prescribe antibiotics in the setting of a negative urinalysis and culture.

Despite established guidelines, practice patterns varied greatly amongst pediatricians at our institution. Harmonized, consolidated guidelines between pediatricians and pediatric urologists would improve patient care and efficiency of the healthcare system.

## Data Availability

The original contributions presented in the study are included in the article/Supplementary Material, further inquiries can be directed to the corresponding author.
